# From Industry 5.0 to Forestry 5.0: Bridging the gap with Human-Centered Artificial Intelligence

**DOI:** 10.1007/s40725-024-00231-7

**Published:** 2024-09-11

**Authors:** Andreas Holzinger, Janine Schweier, Christoph Gollob, Arne Nothdurft, Hubert Hasenauer, Thomas Kirisits, Carola Häggström, Rien Visser, Raffaele Cavalli, Raffaele Spinelli, Karl Stampfer

**Affiliations:** 1 University of Natural Resources and Life Sciences Vienna, Vienna, Austria; 2 Swiss Federal Institute for Forest, Snow and Landscape Research (WSL), Zurich, Switzerland; 3 Swedish University of Agricultural Sciences, Uppsala, Sweden; 4 University of Canterbury, Christchurch, New Zealand; 5 University of Padova, Padua, Italy; 6 CNR IBE, Sesto Fiorentino, Florence, Italy

**Keywords:** Artificial Intelligence, Human-Centered AI, Forestry 5.0, Forest management

## Abstract

**Purpose of the Review:**

Recent technological innovations in Artificial Intelligence (AI) have successfully revolutionized many industrial processes, enhancing productivity and sustainability, under the paradigm of Industry 5.0. It offers opportunities for the forestry sector such as predictive analytics, automation, and precision management, which could transform traditional forest operations into smart, effective, and sustainable practices. The paper sets forth to outline the evolution from Industry 5.0 and its promising transition into Forestry 5.0. The purpose is to elucidate the status of these developments, identify enabling technologies, particularly AI, and uncover the challenges hindering the efficient adoption of these techniques in forestry by presenting a framework.

**Recent Findings:**

However, the gap between potential and practical implementation is primarily due to logistical, infrastructural, and environmental challenges unique to the forestry sector. The solution lies in Human-Centered AI, which, unlike the Industry 4.0 paradigm, aims to integrate humans into the loop rather than replace them, thereby fostering safe, secure, and trustworthy Human-AI interactions.

**Summary:**

The paper concludes by highlighting the need for Human-Centered AI development for the successful transition to Forestry 5.0 – where the goal is to support the human workers rather than substituting them. A multidisciplinary approach involving technologists, ecologists, policymakers, and forestry practitioners is essential to navigate these challenges, leading to a sustainable and technologically advanced future for the forestry sector. In this transformation, our focus remains on ensuring a balance between increased productivity, nature conservation and social licence, worker safety and satisfaction.

## Introduction

**Fig. 1:**
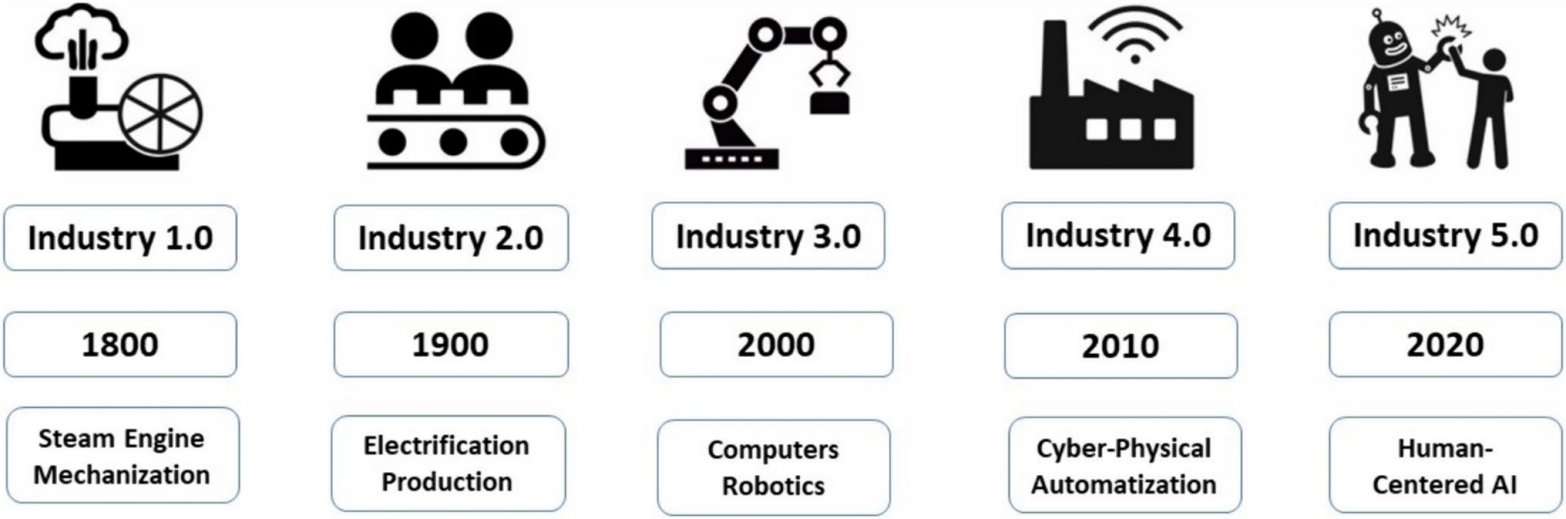
The industrial revolution from Steam Engines to human-centered AI

Industry 5.0, also known as the Fifth Industrial Revolution (5IR), represents the next phase in the evolution of the industrial sector (see Fig. 1), and is at the top of the agenda of the European Union [1]. Whilst Industry 4.0 was driven by the great successes in Artificial Intelligence (AI) and the associated urge to automate everything to replace humans, Industry 5.0 propagates a human-centred AI approach that no longer wants to replace humans but rather augment them making automation a “team-player” in joint human-AI activities [2]. Human-centered AI puts humans in control of AI, aligning AI with human intelligence, social values, ethical principles, and legal requirements to ensure secure, safe, trustworthy and controllable AI [3, 4]. In the context of forest engineering this might be of relevance when for example the selecting single trees for specific purposes and respective management. We provide a brief overview about (i) the development from industry 1.0 to 5.0; (ii) the evolution from digital computers to digital transformation, and (iii) some fundamentals of machine learning – the workhorse of AI. The core of the review is to show use cases to be further developed in human-centered AI within smart forestry and to provide a framework of Forestry 5.0.

## From Steam to AI: The Industrial Revolution from I1.0 to I5.0

Embarking on a journey from the dawn of steam power to the forefront of artificial intelligence, Fig. 1 unveils the transformative industrial revolutions from Industry 1.0 to Industry 5.0.

**Industry 1.0,** the first industrial revolution (late eighteenth century to early nineteenth century) was characterized by the replacement of human power (mechanization) [5, 6]. **Industry 2.0** (late nineteenth century to early twentieth century) initiated by the transition from steam energy to electric energy [7], enabled further innovations such as assembly production lines for mass production [8]. **Industry 3.0** (mid-twentieth century) was marked dominantly by electronics, particularly digital information technology (IT), which mainly replaced human operators by computer-controlled machines [9, 10]. **Industry 4.0** (early twenty-first century and ongoing) is based on the success of the Internet. It is driven by integrated, automated, and connected systems, bringing together physical and digital technologies and focusing on completely autonomous machines to optimize production [11, 12]. Machines, systems, and products can connect and communicate in real-time, enhancing efficiency and promoting effective decision-making, which is the concept of a smart factory [13, 14]. Managing the sheer amount of data available through those internet-connected systems requires dedicated high-power tools, such as machine learning (ML) algorithms which learn from fused data, becoming capable to improve operations, predict outcomes, and even make decisions, in a fully automatic way, without human intervention [15, 16]. **Industry 5.0** (current and ongoing) focuses on the collaboration between humans and AI by placing humans at the centre of the decision-making process [17–19]. It follows the human-centered AI approach, which augments human capabilities instead of replacing humans [20, 21]. The role of a human-in-the-loop [22] is to guarantee that decision-making integrates the goals, understanding, creative capacities and common sense of humans [23].

Alan Turing created the groundwork for modern digital computers [24] and his 1936 work [25] on computable numbers introduced the “universal machine” (today called “Turing machine”), which could imitate any machine with the correct inputs and instructions. All modern digital computers are based on this general-purpose machine concept.

The invention of the transistor in 1947 lowered computer size, cost, and energy consumption while increasing dependability and computing power, boosting digital computer success enormously. The three success concepts of digital computers include scalability, replicability and connectivity, and the breakthrough came with the availability of “big data” and computing power [26–28].

Digital transformation is the process of incorporating digital technology into all aspects of a business or society, resulting in significant changes to how organizations function and provide benefits to consumers [29]. The process goes beyond just converting processes into digital formats or setting up computer systems. Instead, it requires a comprehensive reconsideration of business models, strategies, and customer interactions in response to the fast-paced advancements in digital technologies. The goal is to offer customers seamless and integrated experiences across multiple channels, enhancing their engagement and loyalty. Mobile applications, artificial intelligence chatbots, and customized recommendations all play a role in enhancing consumer experiences. The economic impact of digital transformation is significant. By improving operational efficiency, companies can lower costs and boost productivity, hence promoting economic growth. Additionally, digital transformation can also contribute to environmental sustainability [30].

Digital transformation fosters innovation, resulting in the emergence of novel industries and employment opportunities. Furthermore, improved data analysis can lead to more effective policymaking and resource allocation in public administration, enhancing social welfare. On a broader level, digital transformation can help address major societal challenges. For example, digital technologies are crucial in the transition to a more sustainable economy, through improvements in energy efficiency, the growth of renewable energy, and the development of circular business models [31].

Experts universally agree that the progress in artificial intelligence is the main driving force behind digital transformation, with machine learning acting as its core operational mechanism. Prominent instances of this achievement include the highly prosperous large language models, popular known as ChatGPT created by OpenAI, or Bard developed by Google, and Aleph Alpha, which seeks to establish a leading position in European AI advancement, prioritizing ethical and transparent AI in accordance with European standards and legal viewpoints [32].

Today the widespread success of machine learning has led to AI gaining immense popularity in almost every field of application [33]. AI has a rich history in computer science and focuses on the overarching objective of developing “intelligent” machines [24]. However, the concept of intelligence lacks a precise definition, making it challenging to measure [34, 35]. The foundational principles of AI were initially developed in 1956 by a group of computer scientists during a workshop held at Dartmouth College. The session aimed to establish highly ambitious objectives for AI: *“The study is to proceed on the basis of the conjecture that every aspect of learning or any other feature of intelligence can in principle be so precisely described that a machine can be made to simulate it. An attempt will be made to find how to make machines use language, form abstractions and concepts, solve kinds of problems now reserved for humans, and improve themselves *[36]*”.*

Consequently, it is imperative to recognize that AI is an overarching “umbrella” term denoting intelligent machine learning based systems capable of executing tasks traditionally necessitating human cognition, including problem-solving, and decision-making. ML as the principal mechanism within AI, necessitates a precise foundational understanding. ML types can be systematically classified according to a) their learning approach, b) the tasks they undertake, and/or c) their structural and behavioural characteristics.

- *ML categorization based on learning approach*
  Supervised Learning: The model is trained on a labelled dataset, which means the algorithm is provided with input–output pairs. The aim is to learn a mapping ${\varvec{y}}={\varvec{f}}({\varvec{x}})$ from inputs to outputs. Examples of this category are Linear Regression, Decision Trees, and Neural Networks.
  Unsupervised Learning: The model is trained on an unlabelled dataset, where the algorithm tries to identify patterns or relationships in the data without explicit guidance. Examples used in forestry include Clustering (K-Means), Association (Apriori), and Principal Component Analysis (PCA).
  Semi-Supervised Learning: This is an intermediate type that finds its place between supervised and unsupervised learning. It uses both labelled and unlabelled data for training: often a small amount of labelled data and a large amount of unlabelled data.
  Reinforcement Learning: In this category, an agent learns by interacting with a selected environment and by receiving rewards or penalties in return for its actions. The goal is to learn a policy that maximizes cumulative rewards over time. Examples of this category are Q-learning, Deep Q Network (DQN), and Policy Gradient methods.
- *ML categorization based on task*
  Classification: Assigning data to predefined categories, e.g., healthy tree vs. infested tree.
  Regression: Predicting a continuous value, e.g., predicting forest fire risk as a function of selected factors, etc.
  Clustering: Grouping similar data points together, e.g., tree density, soil moisture, etc.
  Dimensionality Reduction: Reducing the number of random variables under consideration and obtaining a set of principal variables, e.g. via Principal Components Analysis (PCA).
  Association Rule Mining: Discovering interesting relations between variables in large databases, e.g., via apriori.
- *ML categorization based on model structure or behaviour.*
  Neural Networks & Deep Learning: Multi-layered neural networks, including Convolutional Neural Networks (CNNs) for image tasks, Recurrent Neural Networks (RNNs) for sequential data, Transformer architectures for various tasks, and more.
  Ensemble Learning: Using multiple models and aggregating their outputs for better predictions. Examples: Random Forests, Gradient Boosting Machines (GBM), AdaBoost.
  Bayesian Learning: Based on Bayes theorem, it deals with probability inference. e.g., Naive Bayes, Bayesian Networks.

## Use-Cases for AI in Smart Forestry

Smart Forestry, “intelligent forestry”, or Forestry 4.0 [37] uses a wide variety of technologies including sensors [38], robots [39], cyber-physical systems [40], drones [41] and satellites [42]. We demonstrate that the application of AI-augmented technology can enhance the efficiency and effectiveness of forestry processes, including planning, tree felling, transportation, and reforestation. We illustrate a range of common examples where AI is utilized in forest engineering. In the following, we will also use the overarching term AI here, as the reader now knows that in most cases it is based on machine learning.

### Tree Species Identification

In 1976, Meyers [43] conducted a comprehensive analysis of the existing research on identifying tree species using aerial images and discussed the various elements that influenced the progress of this subject in different types of forests. His vision was further developed and today, AI is used for assessing individual tree detection (ITD), [44] and for characteristics including volume and crown dimensions. Standard techniques include Light Detection and Ranging (LiDAR) used via Airborne Laser Scanning (ALS), Mobile Laser Scanning (MLS), or Terrestrial Laser Scanning (TLS), each offering varying degrees of resolution, coverage, and detail suited to specific forestry analysis needs [45–54]. With regard to forest operations, tree species identification has the potential to increase precision and efficiency in forest management and harvest planning, especially when applying innovative logistics concepts such as the forest warehouse [55, 56], which requires a precise identification of all trees within a stand. Furthermore, tree results of automated tree species identification could support the drafting of accurate, geo-referenced harvesting instructions, or harvesting plans. In fact, the tree identification algorithm could be programmed into the harvester on-board computer for automatically selecting the correct grading strategy for each species, and for drafting accurate production lists. In fact, tree species identification would be a prerequisite for autonomous forestry machines [57].

### Tree Quality Assessment

Additionally, AI techniques would be important for quality assessment of standing timber as part of the tree selection during thinning [58], and potentially increase precision in the sorting of timber [59]. Accordingly, tree quality assessment is prerequisite for autonomous forestry machines and has great potential to support forestry work both as an autonomous function and as decision support.

### Automated Tree Selection

Such automation of tree selection could integrate, mediate and potentially solve the differences that occur in human selection when different actors are involved within the timber harvesting chain. Studies by Spinelli et al. (2016) [60] and by Eberhard and Hasenauer (2021) [61] showed that different agents (e.g. certified foresters, loggers, forestry students etc.) may select different trees for harvesting, even if they will agree for a majority of cases (approximately 70%). While simulations show that such difference may have limited effects on the key stand parameters (e.g. DBH, tree height or standing volume) after 50-years of tree growth [61], the immediate effect on value recovery and site amenity may still be large. For that reason, one may think about stakeholder concertation when selecting trees for harvesting or release; however, that would entail a cumbersome procedure with minimal potential for real-life application. On the other hand, such participatory process could be resorted to when drawing a tree selection algorithm, which could then be smoothly applied in the field, without further need for any cumbersome procedures. In turn, the eventual algorithms could be easily designed for integrating the strong regional focus of most rule-based tree selection schemes, which makes generalization a very elusive target [62].

A sub-set of the tree’s selection applications is that of anchor-tree selection in cable yarding operations. Safe installation of a cable yarder requires careful selection of natural anchors, most often in the form of robust trees or stumps [63]. Anchor selection rules are necessarily simple, for fast application under field conditions, but AI would lend itself to developing better and more reliable rules that could be associated to a smartphone app, so that the user would simply need to input the base cable line parameters (mostly maximum tension at the anchor points) then aim at the forest to find suitable trees or stumps, based on their diameter and the angle that the cable would form when tied to any of them [64]. For tree harvesting in at least Central- and South Europe, forest managers traditionally mark trees prior to removal. Typical examples are thinning and shelter wood cuttings. Thinnings are applied within young to middle aged forest stands aiming to reach a special management target, such as enhancing the biodiversity of a stand, supporting renaturalisation processes [65] or promoting high-quality trees by reducing the within stand competition so that the growth rates per unit area will be concentrated to these high-quality trees [66].

During the life span of a forest rotation, commonly 2 to 3 thinning interventions are applied. However, thinning is expensive and using harvester combined with an automated tree selecting procedure is a substantial factor in reducing the harvesting costs, since it would increase the efficiency of a certified forester for selecting the trees [67].

### Automated Harvesting

Forest engineering encompasses a crucial and groundbreaking aspect: the utilization of autonomous harvesting machinery that integrates various artificial intelligence functions and techniques. (Arbeitsmarke – Ansetzen zum automated forestry).

There is little doubt autonomous machinery will play an important role in forest operations in the future. Many machine functions already have the support of automation, and the implementation of remote control of the machine where an operator can operate a piece of equipment, typically in clear line-of sight, at least is commonly available. Teleoperation is where the operator works from a virtual environment with live video and audio feedback from the machine. Autonomous systems of the future are defined by being able to perform certain functions without direct control of a human operator [68]. This is a key step towards the precision forestry of the future [69•]. One important aspect is that they may cause less soil- and stem damage through the use of a lighter and more compact cab-less machine and reduce the risk of harvesting after catastrophic events by removing the operator from a risky workplace, as it has already been done for remote-controlled feller-bunchers [39, 70].

Li & Lideskog (2021) [71] developed AI-applications to detect stumps and rocks etc. for the application of autonomous forest machines. Yang et al. (2023) [72] focus on navigation autonomy to improve path planning, similar to e.g. Reinhart et al. (2020) [73] who presented a methodology on learning-based path planning for autonomous exploration of subterranean environments using aerial robots; and Nevalainen et al. (2020) [74] who proposed a two-phase on-board process, where tree stem registration produces a sparse point cloud which is then used for simultaneous location and mapping. Hera et al. (2023) explored the feasibility of autonomous forest operations extensively, and underscored the potential in autonomous forestry machinery, e.g. log extraction in the cut-to-length harvesting process whilst minimizing environmental impact [75].

To summarize, smart forestry technologies could enable more efficient and sustainable logging activities. Robots and drones, guided by AI algorithms, can identify which trees are mature enough for harvesting while preserving the younger ones.

### Optimization of Wood Transport and Logistics

The optimization of timber delivery and timber truck routing have been a traditional field of interest for forest engineers for at least two decades (e.g. [76]). The advent of AI offers a unique opportunity to develop a set of powerful, effective and robust tools, which can substantially improve the efficiency of both harvesting and transportation processes, i.e., for:

- *Route Optimization:* One of the most immediate applications of AI in wood transport is route optimization. Machine learning algorithms can analyze multiple variables such as road conditions, traffic patterns, and weather forecasts to identify the most efficient route for timber transportation. Dynamic routing can further adapt to real-time changes, thereby reducing fuel consumption and minimizing travel time [77].
- *Load Optimization:* AI can also be applied to maximize the load-carrying capacity of each transport vehicle. Through computational algorithms, the system can determine the optimal arrangement of logs, considering factors such as weight, volume, and type of wood [78]. This ensures that each trip is as productive as possible, reducing the overall number of trips needed and, consequently, the carbon footprint [79, 80].
- *Harvest Planning Integration:* AI algorithms can synchronize the harvesting plans with the logistics operations. For instance, machine learning models can predict when and where the next batch of wood will be ready for transportation, so as to optimize truck fleet deployment and maximize backhauling opportunities. This predictive capability allows for better planning of transport resources and, in the long term, cost reduction [81].
- *Resilience to Disruptions:* Supply chain disruptions like road closures, vehicle breakdowns, or sudden demand fluctuations can have a significant impact on wood logistics. AI’s predictive analytics and real-time monitoring capabilities enable the rapid identification and mitigation of such issues, making the supply chain more resilient [15, 82, 83].
- *Environmental Impact assessment:* AI models can be trained to optimize routes and loads not just for efficiency, but also for minimizing environmental impact. Algorithms can be designed to prioritize routes that are less likely to cause soil compaction, erosion, or damage to the surrounding ecosystem [83]. That is especially important for in-stand traffic: AI can match water table maps, weather forecasts and log distribution to calculate extraction paths that avoid sensitive terrain, thus minimizing environmental impact and maximizing vehicle mobility. Life-cycle assessments (LCA) can be incorporated into the decision-making process to ensure sustainability [84]. *Water and soil properties detection:* Minimizing ecological disruption is paramount. AI can be programmed to detect areas with high soil moisture [85], to ensure low soil compaction, avoid nutrient depletion, and prevent damage to the remaining stand [86] – a good example here is also the work of Flisberg et al. (2021) [87]. Moreover, LCA can be integrated into AI algorithms to guide more sustainable practices.
- *Energy-Efficient Operations:* through data analytics, AI can recommend energy-efficient operating modes for vehicles or machinery involved in wood harvesting, extraction and transportation, contributing to overall sustainability goals [69, 81, 88].

### Supply Chain Management and Disruptions

Having a supply chain from forest to factory without interruptions is crucial for efficient performance. Here, AI-based modelling and simulating can be employed to optimize logistics, predict demand, and better manage resources [89]. When disruptions occur, such as natural hazards or capacity bottlenecks, AI systems can assist humans, who are stressed in such moments, to adapt the supply chain. Machine learning algorithms can forecast and mitigate these challenges, making logistics more resilient while minimizing negative social and environmental impacts [90].

### Forest Visualization

Having access to high-resolution data allows the development of virtual forests with modern immersive visualization technologies simulating forest environments [91]. Such applications could be used for simulation, education and training. The use of game-based simulations is very promising for boring but legally required training aspects, for example, where repetitive tasks must be drilled [92]. Simulations (via Augmented Reality, or Virtual Reality) are also very helpful for forest management e.g. to improve safety for heavy machinery operators [93]. Simulations can be used for communication among stakeholders but also for the greater public [94].

### Forest Damage Detection and Health Monitoring

AI is used to assess the health status of trees aiming to identify areas that are at risk and to implement preventive measures by equipping sensors and satellites with multispectral and hyperspectral imaging.

For example, AI is used for wildfire identification [33], which is central for environmental degradation, but hard to discover at an early stage. A faster and more accurate detection and set alarm can profoundly support humans to identify risk areas, plan fire prevention measures and/or successfully act. Georgiev et al. (2020) [95] presented an approach for autonomous early fire detection, which is based on a system with high degree of reliability. To provide the autonomous capabilities to the proposed system, they have developed an object detection method, based on a convolutional neural network. To have a better field of view over the observed area, instead of traditional lookout towers and satellite-based monitoring, they used the live video feed from an unmanned aerial vehicle (UAV), which patrolled over the risky area. To make better predictions on the fire probability, they did not use only the optical camera of the UAV, but also an on-board thermal camera. Cyber-physical systems can monitor environmental parameters such as temperature, humidity, and wind speed to assess the risk of forest fires. Moreover, AI is used for early identification of bark beetle infestation [96]. For example, Andresini et al. (2023) [97] explored the achievements of ML to perform inventory mapping of bark beetle infestation hotspots in Sentinel-2 images. Their aim was to produce a prediction of the bark beetle infestation masks. They used an explainable AI technique to study the relevance of spectral information and explain the effect of both self-training and spectral vegetation indices on the mapping decision. Knebel et al. (2022) [98] tested a bark beetle early warning system with audio data, data on pheromones and information for a drought stress assessment of the affected trees, which were all collected and used as a basis for an AI-based analysis.

The human-in-the-loop model is essential for validating AI predictions and refining machine learning models based on expert knowledge. It can predict risks such as bark beetles and fires with high accuracy, but incorporating human-in-the-loop ensures that these predictions align with human intuition and expertise, especially in complex or borderline cases.

### Reforestation

Recently, deforestation has threatened Earth's natural cycles. Drones can efficiently plant seeds over large, deforested areas, guided by AI algorithms that identify the optimal planting locations [99]. The use of drones to spread seed aggregates has been trialed around the world as a means of regenerating tree cover in remote areas. High seed application rates, impressive seed germination rates and lower labour costs can be achieved. In addition, drones equipped with appropriate sensors can be useful in analysing the success of seeds after sowing, from the early germination stage through to canopy development.

Once the location data is captured, artificial intelligence, particularly machine learning algorithms, can be used to process the captured images in the data preparation, feature extraction, model training and prediction stages. The segmentation techniques usually involve the implementation of watershed-based algorithms or convolutional neural networks. The use of machine learning in the characterization of tree parameters improves the speed, accuracy and reliability compared to conventional image processing techniques [100]. A human-in-the-loop expert can adjust these algorithms based on knowledge about local ecosystems, thus ensuring successful reforestation projects [101].

### Fertilizing

AI based fertilizing systems can efficiently identify plants in need for fertilization, drones can later fertilize those specific plants optimizing the fertilization procedure. To this end, aerial images can first be taken with a drone to show the condition of the tree crowns. The application of fertilizers via drones depending on tree health will lead to cost savings in the industry and possibly increase production [102].

## Towards a Framework of Forestry 5.0 with Human-Centered AI

Forestry 5.0 represents the next generation of forest management and practices, leveraging advanced AI technologies to enhance sustainability, productivity, and resilience. This concept builds on the principles of Industry 5.0 [103] and Agriculture 5.0 [104], including human-centered AI [105] as a synergistic approach to reconcile artificial intelligence with human intelligence, addressing human social values, ethical principles and legal requirements to ensure safe, secure and trustworthy AI with the human-in-control.

**Fig. 2:**
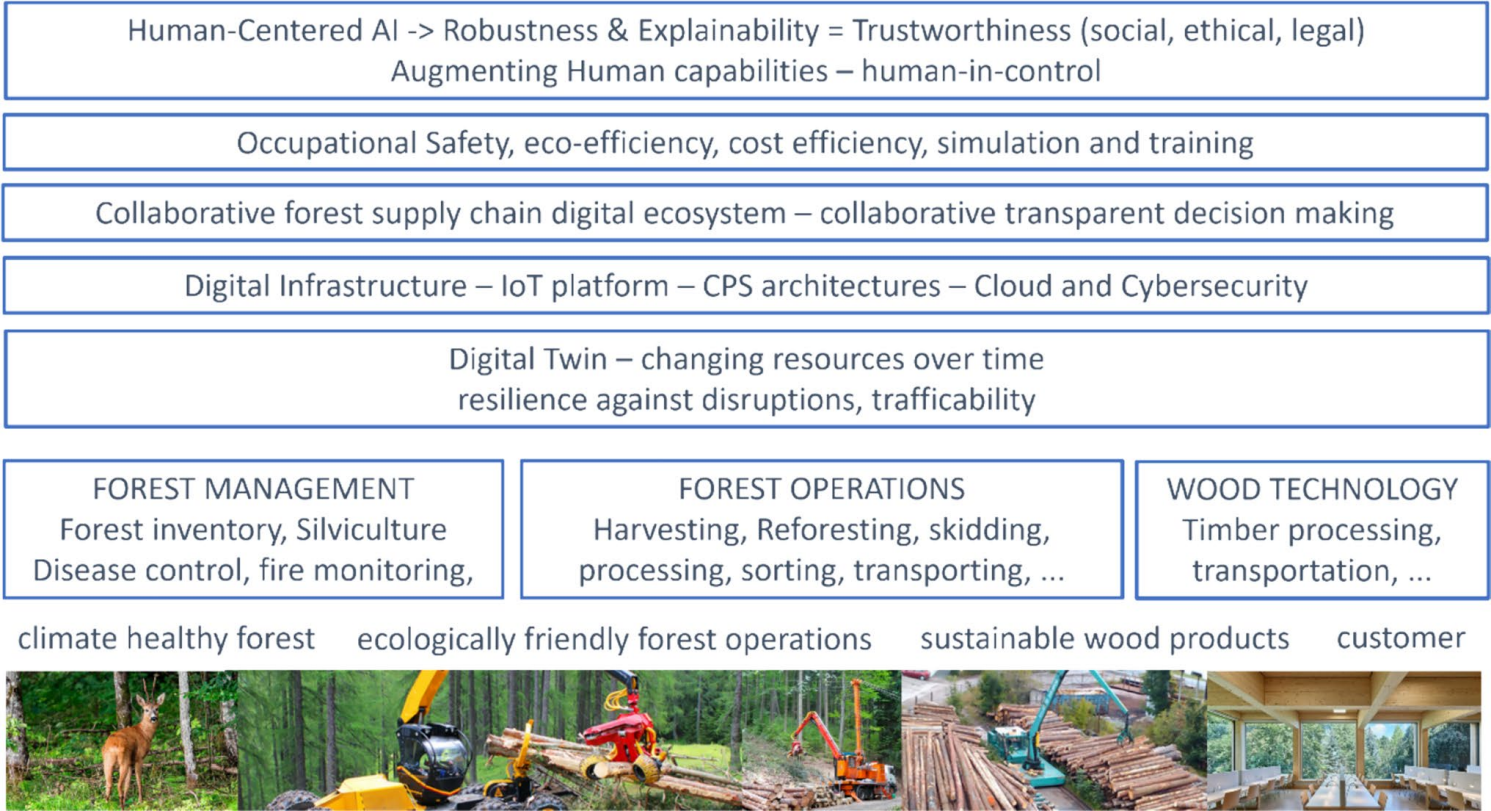
Our envisioned framework for Forestry 5.0

In our Forestry 5.0 framework (Fig. 2) the main goals are to ensure climate healthy forests, ecologically friendly forest operations with sustainable wood products for an economically and ecologically satisfied customer. The three main fields of forestry to achieve these goals include forest management, forest operations and wood technology.

In our view, Forestry 5.0 has the potential to bridge the gap between forest experts and AI experts by fostering their collaboration. We perceive forest experts as individuals engaged daily in, with, or for the forest, and advocate for AI to assist them in their endeavours. Additionally, we acknowledge scientists as forest experts depending on the context. We regard AI experts primarily as computer scientists who specialize in the development, analysis, and application of AI technologies, and we also recognize the diverse range of specialists contributing to the AI field. We see no immediate need to delineate between different types of AI experts, as each brings valuable perspectives and skills to the advancement of AI for Forestry 5.0.

Forest experts are responsible for managing forests in a way that the resilience of future forests, the provision of biodiversity and multiple forest ecosystem services are increased, and ecosystem damage and social and economic effects caused by climate change are reduced or avoided. In view of the complexity of the subject, the multi-objective forest management aims pursued, the growing share of uncertainty and the increasing complexity in decision-making forest experts must tackle needs to be supported through AI experts. In turn, AI experts bring specific expertise about algorithms, machine learning models, data analytics, robotics, and big data analytics, but usually have no forest expertise. In the context of Forestry 5.0, we envision that these two domains collaboratively work together in the following ways:

- **Data Collection and Interpretation:** Forest experts can guide AI experts on what kind of data to collect for meaningful analysis. This might include data on tree growth, species distribution, soil conditions, climate patterns, work procedures and more. AI experts can then design suitable systems for collecting this data efficiently — perhaps using IoT devices or drones — and apply ML- algorithms to interpret the data, extract meaningful trends and ultimately derive useful knowledge.
- **Predictive Modelling:** AI experts can develop predictive models based on the data and insights provided by the forest experts. These models can predict future forest growth, the spread of diseases or pests, the impacts of climate change, or the potential outcomes of different forest management strategies.
- **Intelligent Automation:** AI experts can work with forest experts to develop intelligent automation systems that improve forestry operations. For example, they could design a robotic system for planting or harvesting trees using input from forest experts to ensure the system works effectively and minimally impacts the environment.
- **Education and Training:** Forest experts and AI experts can learn from each other, fostering an exchange of ideas that leads to innovative solutions. Forest experts can gain a better understanding of AI and its potential applications in forestry, while AI experts can deepen their knowledge of forest ecosystems and the challenges they face.
- **Forest Engineering Education:** The increasing penetration of information, communication and artificial intelligence and the interconnection of software and hardware in all forestry processes through digital transformation requires a different way of thinking and acting, especially in the field of engineering education [106]. Lifelong learning and transdisciplinary education are a must, and teaching must include sustainability, resilience and human-centred design, as well as practical courses in data literacy and data management, and experience with human/AI and machine/robot/computer interaction [17].

In summary, the integration of AI in forest management and subsequent supply chains offers a gamut of opportunities for optimization, resilience, and innovation. It enhances traditional practices like tree marking by adding layers of efficiency and sustainability, creating a comprehensive system that benefits both the industry and the natural environment. Bridging the gap between the potential of AI-enhanced forest management and the next phase of human–machine collaboration, it’s clear that the journey from traditional practices to advanced, sustainable forestry requires a nuanced approach. This transition not only promises to augment human capabilities and decision-making but also paves the way for personalized, ethical, and community-engaged forest management strategies, i.e.:

### Human–Machine Collaboration

Embrace and promote collaboration between forest workers and advanced technologies. Use AI and automation to assist in tasks that are dangerous or strenuous for humans, such as felling trees or fighting forest fires. That will free humans from the burden of those menial tasks and allow them to focus on other more complex tasks that require judgement, decision-making, and complex motor control.

### Personalized Forestry Management

Use data analytics, AI, and machine learning to tailor forest management practices to specific forests or even specific areas within a forest. This could involve optimizing for different goals like timber production, biodiversity, carbon sequestration, or recreation based on local conditions and societal needs – essentially one further step in the direction of precision forestry.

### Sustainable Practices

Forestry 5.0 should incorporate a strong focus on sustainability, making use of intelligent technologies to optimize the use of resources, reduce waste, and enhance the health and resilience of forests. This could involve techniques such as precision forestry, where data and digital tools are used to precisely manage forests at a granular level, improving sustainability and productivity.

### Reskilling and Upskilling

Invest in education and training to equip the forestry workforce with the skills needed to work effectively with the new technologies. This could involve skills in data analysis, remote sensing, operating drones or other automated equipment, and managing complex decision-making processes.

### Health and Wellbeing

Use AI not only to optimize operations but also to improve the working conditions and experiences of forest workers. This could involve using AI to improve safety, for example by predicting and preventing accidents [107], or to provide decision-support tools to assist workers in complex tasks, and to manage emergencies whenever prevention would fail. Emergency management is an especially difficult task, because effective decision-making can be hindered by cognitive and/or emotional overload, and that is when automated assistance can become a lifesaver.

### Community Engagement

Utilize digital platforms and technologies to increase engagement with local communities, stakeholders, and citizens. This could involve sharing data and insights about forest health, soliciting input on management decisions, or using citizen science to gather data and insights. Furthermore, public digital platforms could help explain to the general public what they often perceive as “deforestation” or “tree murder”, thus relieving public concern and removing wrong perceptions around forest management.

### Ethics, Inclusivity and Legal Issues

Ensure that the benefits of Forestry 5.0 are distributed equitably and that the transition is managed in a way that is fair to workers, local communities, and indigenous groups. This could involve education and retraining programs, as well as policies to promote equitable access to the benefits of new technologies. Incorporating the importance of legal considerations, particularly under the European AI Act, it’s imperative to navigate the transition to Forestry 5.0 with a keen eye on compliance and ethical standards. This involves not only ensuring equitable distribution of benefits and fair management practices but also adhering to legal frameworks that govern AI usage, thereby safeguarding the rights of workers, communities, and the environment while fostering innovation and trust in new technologies [107, 108], which requires explainable AI [109] with human-centered visualization techniques [110].

## Discussion: Current State and Future Prospects of Forestry 5.0

Forestry 5.0 is an emerging field with varying levels of maturity across its components. In the following we discuss a few issues on how far we are from practical implementation, highlighting ongoing research, early implementations, bottlenecks, and potential solutions.

### Research Level Applications in AI

Research Stage: AI models for predicting tree growth, disease spread, and forest health are in active research. AI models are being developed to analyze satellite imagery and sensor data for better forest management insights.

Challenges: Developing models that can generalize across diverse forest ecosystems and handle the vast complexity of ecological data.

### Early Implementations in IoT, Robotics and Data Analytics

1. Internet of Things (IoT).
  Practical Implementations: IoT devices and sensor networks are being deployed in pilot projects for real-time monitoring of environmental conditions, wildlife tracking, and resource management.
  Challenges: Scalability, data integration from diverse sources, and ensuring reliable and long-term operation in harsh environmental conditions.
2. Robotics and Automation.
  Practical Implementations: Drones are used for aerial surveys, monitoring forest health and reforest. Autonomous ground vehicles are in development for maintenance tasks.
  Challenges: High costs, technical complexity, and ensuring the safety and reliability of autonomous systems in forest environments.
3. Data Analytics:
  Practical Implementations: Data analytics platforms are being used to process and interpret large datasets from various sources, aiding in decision-making and strategic planning.
  Challenges: Data integration, ensuring data quality, and the need for advanced analytical tools capable of handling large volumes of complex data.

### Bottlenecks and Possible Solutions

#### Technological Integration

Bottlenecks: Integrating diverse technologies into a cohesive system is challenging due to compatibility issues and the complexity of forest ecosystems.

Solutions: Developing standardized protocols for data exchange, investing in interoperable technologies, and fostering collaborations between technology providers and forestry experts.

#### Cost and Accessibility

Bottlenecks: High costs of advanced technologies and lack of accessibility for small-scale foresters.

Solutions: Research into cost-effective alternatives, government subsidies, and public–private partnerships to lower costs and increase accessibility.

#### Regulatory Frameworks

Bottlenecks: Lack of clear regulations and policies supporting the deployment of advanced AI technologies in forestry.

Solutions: Advocacy for policy development, interdisciplinary collaboration to shape regulations, and pilot projects to demonstrate the benefits and safety of new technologies.

#### Skill Development

Bottlenecks: Insufficient training and expertise among forestry professionals to use advanced technologies.

Solutions: Developing specialized training programs, integrating AI education into forestry curricula, and offering continuous professional development opportunities.

Whilst significant progress has been made towards realizing Forestry 5.0 already, the future journey involves overcoming substantial technical, economic, and regulatory challenges. Through focused scientific research and collaboration among stakeholders, the practical implementation of Forestry 5.0 can be accelerated, paving the way for a more sustainable and efficient forestry sector.

## Conclusions

In conclusion, the fusion of AI and forest expertise pursued by Forestry 5.0 can not only alleviate mundane tasks but also contribute to the broader goals of sustainable forest management. The application of Industry 5.0 principles to the forestry sector is a crucial transformative step. The convergence of forestry expertise with advanced technologies will bring forth a new era of sustainable forestry practices, which will better achieve all the environmental, economic and ecological goals of forestry – in line with social, ethical and legal issues. Forestry 5.0 has the potential to revolutionize the forest industry, facilitating *precision management* of forest and non-forest resources, enhancing working productivity and reducing undesired environmental impacts. This is most crucial when fighting climate change. This transformation is underpinned by a human-centered approach to artificial intelligence, where technology serves to augment human skills and knowledge rather than replacing them. By leveraging the strengths of both humans and machines, Forestry 5.0 can empower forest experts to make data-driven decisions, improve operational efficiency, and promote sustainable practices. This shift towards Forestry 5.0 will not only address the pressing challenges facing the forestry sector but also pave the way for a future where human ingenuity and technological innovation will work in harmony to sustain and protect our valuable forest ecosystems.

***Our wish for the future is AI fairness, open science, and open data — AI ecosystems for the benefit of all people on our planet.***

## Data Availability

No datasets were generated or analysed during the current study.
